# Sodium-Glucose Cotransporter-2 Inhibitors for Treatment of Nonalcoholic Fatty Liver Disease: A Meta-Analysis of Randomized Controlled Trials

**DOI:** 10.3390/metabo11010022

**Published:** 2020-12-30

**Authors:** Alessandro Mantovani, Graziana Petracca, Alessandro Csermely, Giorgia Beatrice, Giovanni Targher

**Affiliations:** Section of Endocrinology, Diabetes and Metabolism, Department of Medicine, University of Verona, 37126 Verona, Italy; alessandro.mantovani@univr.it (A.M.); grazianapetracca1@gmail.com (G.P.); csermelyale@gmail.com (A.C.); giorgiabeatricejb@gmail.com (G.B.)

**Keywords:** SGLT-2 inhibitors, type 2 diabetes mellitus, nonalcoholic fatty liver disease, NAFLD, nonalcoholic steatohepatitis, nonalcoholic steatohepatitis (NASH)

## Abstract

Recent randomized controlled trials (RCTs) tested the efficacy of sodium-glucose cotransporter-2 (SGLT-2) inhibitors to specifically treat nonalcoholic fatty liver disease (NAFLD). We systematically searched three electronic databases (up to 31 October 2020) for identifying placebo-controlled or head-to-head RCTs that used SGLT-2 inhibitors for treatment of NAFLD. No published RCTs with paired liver biopsy data were available for the meta-analysis. Primary outcome measures were changes in serum liver enzyme levels and liver fat content on imaging techniques. Overall, we included a total of twelve RCTs testing the efficacy of dapagliflozin (*n* = six RCTs), empagliflozin (*n* = three RCTs), ipragliflozin (*n* = two RCTs) or canagliflozin (*n* = one RCT) to specifically treat NAFLD for a median period of 24 weeks with aggregate data on 850 middle-aged overweight or obese individuals with NAFLD (90% with type 2 diabetes). Compared to placebo/reference therapy, treatment with SGLT-2 inhibitors significantly decreased serum alanine aminotransferase (weighted mean differences (WMD): −10.0 IU/L, 95%CI −12.2 to −7.79 IU/L; *I^2^* = 10.5%) and gamma-glutamyltransferase levels (WMD: −14.49 IU/L, 95%CI −19.35 to −9.63 IU/L, *I^2^* = 38.7%), as well as the absolute percentage of liver fat content on magnetic resonance-based techniques (WMD: −2.05%, 95%CI −2.61 to −1.48%; *I^2^* = 0%). In conclusion, SGLT-2 inhibitors seem to be a promising treatment option for NAFLD.

## 1. Introduction

Nonalcoholic fatty liver disease (NAFLD) has reached epidemic proportions in many parts of the world, and is estimated to affect up to ~70–80% of people with type 2 diabetes mellitus (T2DM) [1,2]. Substantial evidence shows that the coexistence of T2DM and NAFLD synergistically increases the risk of developing not only the more severe histologic forms of NAFLD (nonalcoholic steatohepatitis [NASH], cirrhosis and hepatocellular carcinoma), but also the risk of developing cardiovascular disease and other chronic complications of diabetes [3,4,5]. Therefore, early recognition of NAFLD and monitoring for NASH with advanced fibrosis in people with T2DM are crucial. The coexistence of NASH in a subject with T2DM should call for taking advantage of glucose-lowering agents with proven efficacy to improve cardiometabolic health and prevent liver disease progression [6]. 

To date, there are no approved drugs to specifically treat NAFLD or NASH. Of all glucose-lowering drugs, pioglitazone is the best-studied pharmacological drug in NASH. Although there is evidence that long-term use of pioglitazone in individuals with biopsy-proven NASH has beneficial effects on serum liver enzyme levels, liver fat content and histological resolution of NASH amongst individuals with and without T2DM, this drug may have some side-effects, such as moderate weight gain, fluid retention and risk of distal bone fractures (mostly in post-menopausal women) [7,8]. Promising results on liver fat and histological resolution of NASH have recently been reported also in some phase-2 RCTs using glucagon-like peptide-1 receptor agonists, such as liraglutide and semaglutide [9,10].

Sodium-glucose cotransporter-2 (SGLT-2) inhibitors are a novel class of oral glucose-lowering drugs approved for treatment of T2DM. By reducing the renal capacity to reabsorb filtered glucose, SGLT-2 inhibitors increase renal glycosuria and osmotic diuresis, thereby improving glucose control and exerting additional beneficial effects, such as weight loss and the lowering of blood pressure [11]. Recent large randomized controlled trials (RCTs) on SGLT-2 inhibitors have consistently shown that these glucose-lowering drugs also exert favorable long-term effects on risk of major cardiovascular events, including worsening of heart failure and deterioration of nephropathy, in people with T2DM [12,13,14,15,16]. 

As SGLT-2 inhibitors improve not only glycemic control, but also body weight and blood pressure, a number of observational cohort studies and RCTs have recently examined the possible beneficial effects of this novel class of glucose-lowering medications in individuals with T2DM and NAFLD [7,17,18]. 

The purpose of our meta-analysis was to examine the published data of placebo-controlled or head-to-head RCTs, which tested the efficacy and safety of various SGLT-2 inhibitors to specifically treat NAFLD in individuals with or without established T2DM.

## 2. Results

Supplementary Figure S1 summarizes the results of the literature research and study selection. We initially found 14 potentially eligible RCTs from three large electronic databases prior to 31 October 2020 [19,20,21,22,23,24,25,26,27,28,29,30,31,32]. After examining the full text of these publications, we excluded two studies [31,32] for reasons mainly due to unsatisfactory study design (as specified in Supplementary Table S1). Therefore, a total of twelve RCTs (seven placebo-controlled studies [19,21,23,24,25,26,28] and five active-controlled studies [20,22,27,29,30]) were considered eligible for inclusion in the meta-analysis and were assessed for quality. 

The main characteristics of the included RCTs are summarized in Supplementary Table S2. In total, there were 850 middle-aged overweight or obese individuals with NAFLD (59% men; mean (±SD) age 57 ± 6 years; mean body mass index 31 ± 2 kg/m^2^; mean alanine aminotransferase (ALT) 41 ± 11 IU/L; mean aspartate aminotransferase (AST) 31 ± 7 IU/L; mean gamma-glutamyltransferase (GGT) 59 ± 17 IU/L), who were followed-up for a median period of 24 weeks (interquartile range: 21–25 weeks). Among these individuals, 414 were randomly assigned to either placebo or reference therapy, whereas 436 were randomly assigned to active treatment with dapagliflozin (*n* = 6 RCTs [19,22,24,25,27,30]), empagliflozin (*n* = 3 RCTs [21,26,28]), ipragliflozin (*n* = 2 RCTs [20,29]) or canagliflozin (*n* = 1 RCT [23]) in order to specifically treat NAFLD. As shown in Supplementary Table S2, the vast majority of the eligible RCTs (*n* = 11 studies [19,20,21,22,23,24,25,26,27,29,30]) included patients with NAFLD and T2DM (*n* = 760, 90% of total participants), whereas only one RCT [28], involving 90 individuals, was conducted in patients without T2DM. Two RCTs [19,27] included international cohorts of individuals with NAFLD, six RCTs [20,21,25,28,29,30] were carried out in Asia (Japan, South Korea, India and Iran), three RCTs [22,24,26] were carried out in Europe (Germany and Sweden) and one RCT was carried out in the United States [23]. In all eligible RCTs, the diagnosis of NAFLD was based on imaging techniques, such as Fibroscan^®^ with associated with controlled attenuation parameter (CAP) [25,28,29], computed tomography [20,30], magnetic resonance imaging-proton density fat fraction (MRI-PDFF) or magnetic resonance spectroscopy (MRS) [19,21,22,23,24,26,27]. No published RCTs with paired liver biopsy data were currently available for the meta-analysis. In all eligible RCTs, SGLT-2 inhibitors were usually well tolerated and had a comparable adverse event profile to placebo or reference therapy, except for a higher frequency of genitourinary infections. In Supplementary Table S3, the risk of bias for each eligible RCT assessed by the Cochrane Collaboration’s tool is summarized, which includes seven potential sources of bias. For each domain, we categorized each RCT into three categories: low, unclear, or high risk of bias.

**Figure 1:**
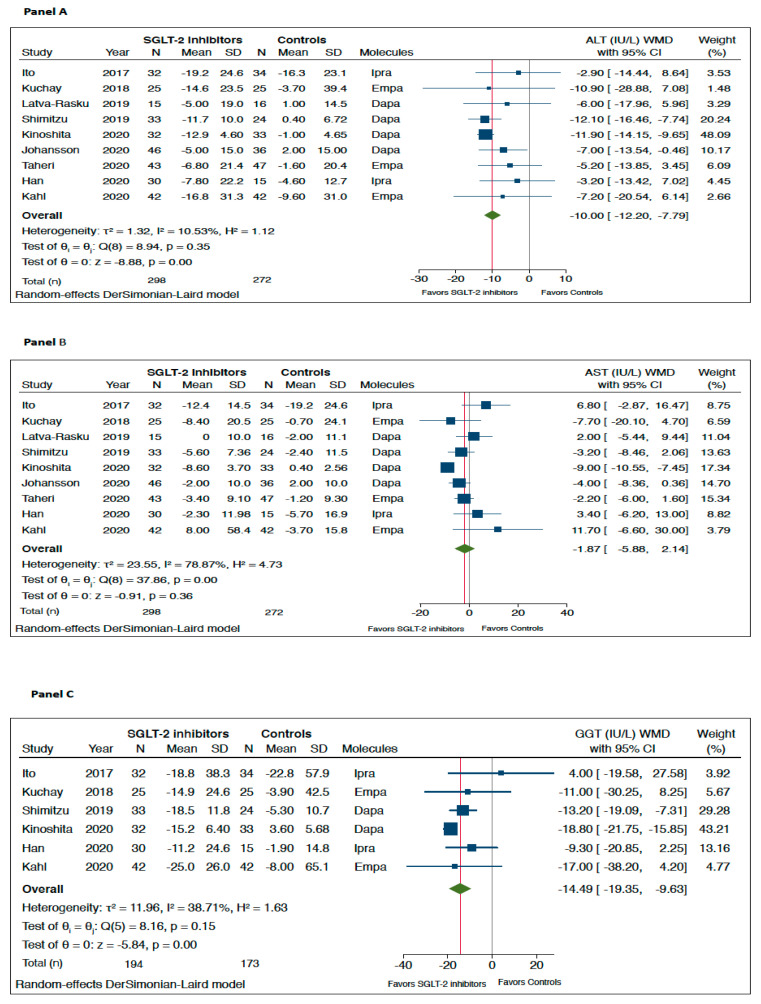
Forest plot of the effect of SGLT-2 inhibitors on serum liver enzyme levels (i.e., serum alanine aminotransferase (ALT) (*n* = 9 randomized controlled trials (RCTs), panel **A**), aspartate aminotransferase (AST) (*n* = 9 RCTs, panel **B**), and gamma-glutamyltransferase (GGT) (*n* = 6 RCTs, panel **C**)) as compared with placebo or reference therapy. The pooled (green diamond) and individual effect sizes for RCTs included were expressed as weighted mean difference (WMD) and 95% confidence intervals (CI). Note: If not available, the SDs of the weighted mean difference were estimated using a specific formula (as reported in the Methods section).

As shown in Figure 1, when compared to placebo/reference therapy, treatment with SGLT-2 inhibitors significantly decreased the levels of serum ALT (panel A: *n* = 9 RCTs; pooled weighted mean differences (WMD): −10.0 IU/L, 95%CI −12.2 to −7.79 IU/L; *I^2^* = 10.5%) and serum GGT (panel C: *n* = 6 RCTs; pooled WMD: −14.49 IU/L, 95%CI −19.35 to −9.63 IU/L; *I^2^* = 38.7%). Serum AST levels did not differ significantly between the two arms of treatment (panel B: *n* = 9 RCTs; pooled WMD: −1.87 IU/L, 95%CI −5.88 to 2.14 IU/L; *I^2^* = 78.9%).

**Figure 2:**
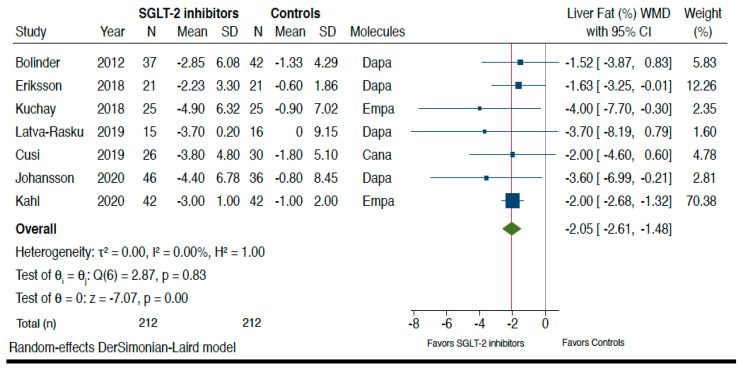
Forest plot of the effect of SGLT-2 inhibitors on the absolute percentage of liver fat content assessed by magnetic resonance-based techniques (*n* = 7 RCTs using either magnetic resonance imaging-proton density fat fraction (MRI-PDFF) or magnetic resonance spectroscopy (MRS)). The effect size was expressed as weighted mean difference (WMD) and 95% confidence intervals for all RCTs included. Note: If not available, the SDs of the weighted mean difference were estimated using a specific formula (as reported in the Methods section). In the study of Bolinder et al. [19], the investigators reported only the placebo-corrected difference in mean percent MRI-PDFF with dapagliflozin.

Figure 2 shows the forest plots and pooled estimates of the effect of different SGLT-2 inhibitors on liver fat content assessed by magnetic resonance-based techniques. Overall, in the seven RCTs included in this analysis, the pooled mean relative percent changes of liver fat content in those treated with SGLT-2 inhibitors and those treated with placebo/reference therapy at the end of the trials were −29% vs. −5.8%, respectively. As shown in the figure, when compared to placebo or reference therapy, treatment with SGLT-2 inhibitors was associated with a significant improvement in the absolute percentage of liver fat content assessed by MRI-PDFF or MRS (*n* = 7 RCTs; pooled WMD: −2.05%, 95%CI −2.61 to −1.48%; *I^2^* = 0%; Z-test for overall effect = −7.07, *p* < 0.0001). 

Table 1 summarizes the pooled estimates of the effect of SGLT-2 inhibitors on liver fat content (not only detected by magnetic resonance imaging—as also previously reported in Figure 2—but also by either CAP on Fibroscan^®^ or computed tomography), as well as on liver stiffness measurement (LSM) assessed with vibration-controlled transient elastography (Fibroscan^®^). In line with the results already reported in Figure 2 where seven RCTs were available for the pooled primary analysis, treatment with SGLT-2 inhibitors also showed a small improvement in liver fat content when assessed either by CAP on Fibroscan^®^ (*n* = 3 RCTs; WMD: −13.9 dB/m, 95%CI −30.1 to +2.20 dB/m; *I^2^* = 43.7%; Z-test for overall effect = 1.69, *p* = 0.089) or by the liver-to-spleen attenuation ratio on computed tomography (*n* = 2 RCTs). It is known that the Hounsfield Unit attenuation of liver on computed tomography scans is usually higher than the spleen; when this ratio is reversed, this can be used to diagnose the presence of hepatic steatosis. Liver-to-spleen attenuation ratio <1.0 can be used effectively to diagnose the presence of hepatic steatosis. Finally, as also shown in Table 1, when compared to placebo/reference therapy, treatment with SGLT-2 inhibitors tended to improve LSMs assessed by Fibroscan^®^ (*n* = 2 RCTs; WMD: −0.65 kPa, 95%CI −1.48 to +0.20 kPa; *I^2^* = 14.0%; Z-test for overall effect = 1.48, *p* = 0.097).

We tested for the possibility of excessive influence of individual RCTs using an influence test that eliminated each of the included RCTs one at a time. Notably, eliminating each of the eligible RCTs from the analysis did not have any significant effects on changes both in serum liver enzymes and in the absolute percentage of liver fat content, assessed by MRI-PDFF or MRS (data not shown).

As summarized in Supplementary Figure S2, when compared to placebo or reference therapy, treatment with SGLT-2 inhibitors was associated with a significant reduction in body weight (panel A: *n* = 9 RCTs; pooled WMD: −3.74 kg, 95% CI −2.56 to −4.91 kg; *I^2^* = 0%), along with a small improvement in hemoglobin A1c levels (panel B: *n* = 7 RCTs; pooled WMD: −0.19%, 95% CI −0.08 to −0.30; *I^2^* = 11.9%). 

As reported in Supplementary Figure S3, the rank correlation Begg’s test did not show any statistically significant asymmetry of the funnel plots of the RCTs examining the effect of SGLT-2 inhibitors on changes in serum ALT levels and MRI-assessed liver fat content (*p*-values = 0.677 and 0.881, respectively), thereby suggesting that publication bias was unlikely.

## 3. Discussion

Compared with the narrative or systematic review articles that have been recently published on this topic [6,7,17,18,33,34], to the best of our knowledge, this is the first comprehensive and updated meta-analysis of placebo-controlled or active-controlled RCTs that used various SGLT-2 inhibitors for treatment of NAFLD. 

Our meta-analysis includes a total of twelve RCTs (seven placebo-controlled and five active-controlled RCTs) testing the efficacy of dapagliflozin (*n* = six RCTs), empagliflozin (*n* = three RCTs), ipragliflozin (*n* = two RCTs) or canagliflozin (*n* = one RCT) to specifically treat NAFLD for a median of 24 weeks with aggregate data on 850 middle-aged overweight or obese individuals with NAFLD (the vast majority of whom had coexistent T2DM, i.e., 90% of total). In all eligible RCTs, the diagnosis of NAFLD was based on different imaging techniques (mostly magnetic resonance-based techniques). Currently, no RCTs with paired liver biopsy data for the diagnosis of NAFLD were available in the literature for the meta-analysis.

When compared to placebo/reference therapy, treatment with SGLT-2 inhibitors for a median of 24 weeks was associated with significant improvements in serum ALT and GGT levels and, most importantly, in the absolute percentage of liver fat content as assessed by different imaging techniques (*n* = 7 RCTs; pooled WMD: −2.05%, 95%CI −2.61 to −1.48%; *I^2^* = 0%, when assessed by magnetic resonance-based techniques). Treatment with SGLT-2 inhibitors also tended to improve liver stiffness assessed by Fibroscan^®^ (but only two small RCTs were available for this analysis). When compared to placebo/reference therapy, treatment with SGLT-2 inhibitors was also associated with a significant reduction in body weight (~3.5 kg) and also a small improvement in hemoglobin A1c levels (~0.2%). In all included RCTs, SGLT-2 inhibitors were well tolerated with a similar adverse event profile to either placebo or reference therapy, except for greater genito-urinary infections (especially genital mycotic infections). 

From our meta-analysis, it clearly emerges that the major issue in this field of research is the scarcity of high-quality RCTs of sufficient duration with paired liver biopsy data, which is the “reference” method for assessing drug-induced resolution of NASH or changes in individual histological scores of NAFLD. In fact, most of the included RCTs (published until 31 October 2020) are small with a short period of treatment and, most importantly, to date, there are no head-to-head or placebo-controlled RCTs testing the effect of SGLT-2 inhibitors on the histological features of NAFLD. Conversely, strong evidence indicates that SGLT-2 inhibitors have beneficial effects on major adverse cardiovascular and renal outcomes in large RCTs of people with T2DM [12,13,14,15,16]. Recent results from the Dapagliflozin and Prevention of Adverse-Outcomes (DAPA)-heart failure trial also showed a significant risk reduction in worsening heart failure or death from cardiovascular causes with dapagliflozin (at a dose of 10 mg once daily), compared to placebo, among patients with heart failure and a reduced left ventricular ejection fraction, regardless of the presence or absence of T2DM [35]. All these findings may represent an attractive bonus for the long-term use of SGLT-2 inhibitors in individuals with T2DM and NAFLD [36]. That said, animal studies in obese mice have suggested a beneficial effect of SGLT-2 inhibitors on hepatic injury (steatosis, hepatocyte ballooning and, in some cases, also fibrosis), possibly due to a combination of negative energy balance by increased glycosuria and substrate switching towards lipids as a source of energy expenditure [37,38]. Experimentally, there are also emerging data to suggest mechanisms beyond the reduction in body weight and hyperglycemia, and a potential role for the decrease in chronic inflammation and oxidative stress associated with SGLT-2 inhibitor treatment [36]. In addition, in a single-arm, open-label, pilot trial, involving nine Malaysian individuals with biopsy-confirmed NASH and T2DM, Lai et al. reported that a 24-week treatment with empagliflozin (25 mg daily) was associated with some improvements in histologic scores of NASH [31]. However, although this pilot study provides preliminary evidence supporting that empagliflozin might be useful for treatment of NASH, larger placebo-controlled RCTs are needed to assess the efficacy of SGLT-2 inhibitors for treatment of NASH in patients with or without T2DM. With regards to this, a multicenter phase-3 RCT with dapagliflozin (i.e., the Dapagliflozin Efficacy and Action in NASH (DEAN) trial) for treatment of NASH is currently ongoing.

The major strength of our study is the use of a systematic review methodology to identify all relevant RCTs (published up to 31 October 2020) that meet predefined inclusion criteria. In addition, the pooled primary results of our meta-analysis show a low heterogeneity (e.g., *I^2^* = 0% for the effect of SGLT-2 inhibitors on changes in MRI-assessed liver fat content). Our meta-analysis also suggests the possibility of a beneficial class effect of SGLT-2 inhibitors on serum liver enzyme levels and liver fat content in people with NAFLD. However, the current lack of head-to-head RCTs does not allow us to definitely ascertain which of the four SGLT-2 inhibitors tested is the most effective on liver fat content (although looking at Figure 2 the beneficial effect of dapagliflozin, empagliflozin or canagliflozin on the absolute percentage of liver fat content assessed by magnetic resonance-based techniques appears to be essentially comparable). That said, this meta-analysis has some important limitations that merit being mentioned. Firstly, our meta-analysis includes a relatively low number of placebo-controlled and head-to-head RCTs with a small sample size and a short duration of treatment (i.e., a median period of 24 weeks). Secondly, restriction to RCTs might have limited generalizability to “real-world” populations of patients with NAFLD and T2DM. Thirdly, most of the eligible RCTs (*n* = 11 studies) included patients with NAFLD and T2DM (90% of total participants), whereas only one small phase-2 RCT [28] was conducted in Iranian patients without T2DM. Although in this latter RCT treatment with empagliflozin for 24 weeks was associated with significant improvements in serum liver enzyme levels and liver fat content, future large RCTs in non-diabetic individuals with NAFLD are urgently awaited. Fourthly, RCTs with liver histological endpoints as a primary outcome were not available in the literature. Although MRI-PDFF or MRS can provide non-invasive, accurate measures of liver fat content, their efficacy for detecting relevant histological features of NAFLD (i.e., NASH and fibrosis) is somewhat limited [39,40]. In addition, magnetic resonance-based techniques may not be as useful to assess treatment response in patients with NAFLD as previously believed, given that some evidence suggests that improvements in liver fat content measured by MRI do not necessarily translate in improvements in liver histology features (lobular inflammation, hepatocyte ballooning, or fibrosis) in patients with NASH [41]. Thus, RCTs of pharmacologic treatments aimed at improving liver disease severity in NAFLD should always include patients with biopsy-confirmed NASH and fibrosis, which are the two of the strongest histological features of NAFLD associated with an increased risk of developing adverse liver-related and extra-hepatic outcomes [5,42,43,44,45]. For these reasons, the RCTs included in this meta-analysis obtained a fair quality according to the Cochrane Collaboration’s tool for assessing risk of bias. Finally, since there are sex differences in the prevalence, risk factors and clinical outcomes of NAFLD [46], future larger RCTs should be specifically designed to explore sex differences in the response to treatment for NAFLD/NASH with this novel class of glucose-lowering agents.

In conclusion, our meta-analysis is the most comprehensive and updated assessment of placebo-controlled or head-to-head RCTs of adult individuals with NAFLD using various SGLT-2 inhibitors to specifically treat NAFLD. Although it has been shown that SGLT-2 inhibitors significantly improve serum liver enzyme levels and liver fat content assessed by imaging techniques (mostly MRI-PDFF or MRS), no robust data are currently available in the literature with liver histological endpoints as a primary outcome to comment on the efficacy of SGLT-2 inhibitors as a specific treatment for NAFLD or NASH. If these promising results will be confirmed in larger phase-3 RCTs with liver histological endpoints, it is reasonable that SGLT-2 inhibitors will become a suitable treatment option in adult individuals with NAFLD or NASH, especially in those who are obese or have T2DM.

## 4. Materials and Methods 

### 4.1. Registration of Review Protocol

The protocol for this systematic review and meta-analysis was registered in advance on Open Science Framework registries (no: osf.io/axd3b). 

### 4.2. Search Strategy and Study Selection

We performed a systematic review and meta-analysis in accordance with the Preferred Reporting Items for Systematic Reviews and Meta-Analyses (http://www.prisma-statement.org). Eligible studies were identified by systematically searching PubMed, Scopus and ClinicalTrials.Gov databases from the inception date to 31 October 2020 (date of the last research), using the following free text terms: “nonalcoholic fatty liver disease” (OR “NAFLD” OR “nonalcoholic steatohepatitis” OR “NASH”) AND “sodium-glucose cotransporter-2 inhibitors” OR “SGLT-2 inhibitors” OR “SGLT2” OR “dapagliflozin” OR “empagliflozin” OR “canagliflozin” OR “ipragliflozin” OR “ertugliflozin”. Luseogliflozin was not included in the meta-analysis, as the use of this drug is currently approved only in Japan [47]. Eligible searches were limited to placebo-controlled or head-to-head RCTs, in which the diagnosis of NAFLD was based on liver biopsy or imaging techniques, such as ultrasonography, vibration-controlled elastography (Fibroscan^®^) associated with controlled attenuation parameter (CAP), computed tomography, or magnetic resonance-based methods (MRI-PDFF and MRS). Reference lists of relevant papers and previous review articles were hand searched for other relevant studies. Studies enrolling patients with significant alcohol consumption (usually defined as alcohol consumption >20 g/day for women and >30 g/day for men, respectively) or secondary causes of chronic liver disease were excluded. Moreover, non-English-language articles and studies reported only in conference abstracts, unpublished studies, retrospective observational studies or non-randomized interventional studies were excluded. Three investigators (G.B., A.C., and G.P.) independently screened citations and assessed the excluded citations. Two investigators (A.M. and G.T.) independently evaluated full-text articles by applying the inclusion criteria and resolved disagreements by consensus.

### 4.3. Data Extraction and Quality Assessment

We extracted the following information from the eligible RCTs: study characteristics (first author, year of publication, sample size), intervention (type and daily dosages of SGLT-2 inhibitors or active drug comparators), length of the trial, methods used for the diagnosis NAFLD, as well as results for effectiveness and harms outcomes. Specifically, the primary outcome measures of interest were changes in serum liver enzyme levels (alanine aminotransferase (ALT), aspartate aminotransferase (AST) and gamma-glutamyltransferase (GGT)) and in the absolute percentage of liver fat content on imaging techniques (mostly assessed by magnetic resonance-based techniques, i.e., MRI-PDFF or MRS). As secondary outcome measures, we also extracted from the eligible RCTs data on changes in liver stiffness measurement assessed by vibration-controlled liver elastography (Fibroscan^®^), weight loss and changes in hemoglobin A1c levels and, whenever available, information on percentage of withdrawals due to adverse events. We also contacted some corresponding authors of the eligible RCTs in order to obtain additional information for the meta-analysis (as reported in the Acknowledgements section).

Two investigators (A.M. and G.T.) independently evaluated the risk of bias for each RCT. For this purpose, the Cochrane Collaboration’s tool was used [48]. Briefly, this tool evaluates seven potential sources of bias: random sequence generation (selection bias), allocation concealment (selection bias), blinding of participants and personnel (performance bias), blinding of outcome assessment (detection bias), incomplete outcome data (attrition bias), selective reporting (reporting bias), and other bias [48]. For each domain, we categorized each RCT into three categories: low, unclear, or high risk of bias [48].

### 4.4. Data Synthesis and Analysis

The effect sizes were displayed as weighted mean difference (WMD) and 95% confidence intervals (CIs) for the included RCTs reporting the primary outcome measures of interest between patients randomly assigned to the placebo/reference therapy or those randomly assigned to treatment with SGLT-2 inhibitors. The overall estimate of the effect size was computed using a random-effects model [48]. If the outcome measures were reported in median, range, or 25th–75th percentiles, the mean and SD values were estimated using validated formulas [49]. If not available, SDs of the mean differences were estimated using the following formula: SD= [(SD pre-treatment)^2^ + (SD post-treatment)^2^ − (2R × SD pre-treatment × SD post-treatment)]^½^ [50]. Given that the pretest–posttest correlation coefficients (R) were not reported in the eligible RCTs, an R value of 0.5 was assumed in this meta-analysis [48,50]. 

Visual inspection of the forest plots was used to estimate the heterogeneity. The heterogeneity among the included RCTs was also tested by the *I^2^*-statistics. Specifically, the interpretation of the *I^2^*-statistics is as follows: *I^2^*-values of roughly 25% show low heterogeneity, *I^2^*-values of roughly 50% show medium heterogeneity, whereas *I^2^*-values of roughly 75% show high heterogeneity [51]. Publication bias was assessed both by visual inspection of the funnel plots and by the Begg’s rank test [52].

All statistical tests were two sided and used a significance level of *p-*value < 0.05. All statistical analyses were performed using the software STATA^®^ 16.1 with the meta-analysis package (STATA, College Station, TX, USA). 

## Figures and Tables

**Table 1:** Effects of SGLT-2 inhibitors on liver fat content (assessed by magnetic resonance-based techniques (MRI-PDFF or MRS), controlled attenuation parameter (CAP) on Fibroscan^®^ and computed tomography (CT)), as well as on liver stiffness measurement assessed by vibration-controlled transient elastography (Fibroscan^®^).

	WMD (95%CI)	Test *Z* for Overall Effect	Number of RCTs Included	Number of Subjects Assigned to Placebo or Reference Therapy	Number of Subjects Assigned to SGLT-2 Inhibitors	Heterogeneity *I^2^*
Liver fat content						
MRI-PDFF or MRS (%)	−2.05 (−2.61–−1.48)	*Z* = 7.07, *p* < 0.0001	7	212	212	0.0%
Controlled attenuation parameter (dB/m)	−13.9 (−30.1–+2.20)	*Z* = 1.69, *p* = 0.089	3	86	106	43.7%
CT-Liver-to-spleen attenuation ratio	+0.10 (−0.06–+0.23)	*Z* = 1.14, *p* = 0.256	2	67	64	86.2%
Liver stiffness on Fibroscan^®^						
Liver stiffness measurement (kPa)	− 0.65 (−1.48–+0.20)	*Z* = 1.48, *p* = 0.097	2	71	76	14.0%

## Supplementary Materials

The following are available online at https://www.mdpi.com/2218-1989/11/1/22/s1, Figure S1: The PRISMA flow diagram for search and selection processes of the meta-analysis; Figure S2: Forest plot of the effects of SGLT-2 inhibitors on body weight (*n* = 9 RCTs, panel A) and hemoglobin A1c levels (*n* = 7 RCTs, panel B) as compared with placebo or reference therapy. The effect size was expressed as weighted mean difference (WMD) and 95% confidence intervals for all RCTs included. NB: If not available, the SDs of the mean difference were estimated using a specific formula; Figure S3: Funnel plots of standard errors by weighted mean difference (WMD) in serum ALT levels (*n* = 9 RCTs, panel A) and liver fat content assessed by MRI-PDFF or MRS (*n* = 7 RCTs, panel B). *p*-values were assessed by the rank correlation Begg’s test; Table S1: Clinical trials excluded at the eligibility step of the PRISMA diagram; Table S2: Placebo-controlled or active-controlled RCTs of different SGLT-2 inhibitors for treatment of NAFLD (ordered by publication year); Table S3: Risk of bias for each RCT assessed by the Cochrane Collaboration’s tool.

## References

[1] Lonardo A., Bellentani S., Argo C.K., Ballestri S., Byrne C.D., Caldwell S.H., Cortez-Pinto H., Grieco A., Machado M.V., Non-alcoholic Fatty Liver Disease Study Group (2015). Epidemiological modifiers of non-alcoholic fatty liver disease: Focus on high-risk groups. Dig. Liver Dis..

[2] Younossi Z.M. (2019). Non-alcoholic fatty liver disease—A global public health perspective. J. Hepatol..

[3] Younossi Z., Anstee Q.M., Marietti M., Hardy T., Henry L., Eslam M., George J., Bugianesi E. (2018). Global burden of NAFLD and NASH: Trends, predictions, risk factors and prevention. Nat. Rev. Gastroenterol. Hepatol..

[4] Targher G., Lonardo A., Byrne C.D. (2018). Nonalcoholic fatty liver disease and chronic vascular complications of diabetes mellitus. Nat. Rev. Endocrinol..

[5] Mantovani A., Scorletti E., Mosca A., Alisi A., Byrne C.D., Targher G. (2020). Complications, morbidity and mortality of nonalcoholic fatty liver disease. Metabolism.

[6] Budd J., Cusi K. (2020). Role of Agents for the Treatment of Diabetes in the Management of Nonalcoholic Fatty Liver Disease. Curr. Diab. Rep..

[7] Mantovani A., Byrne C.D., Scorletti E., Mantzoros C.S., Targher G. (2020). Efficacy and safety of anti-hyperglycaemic drugs in patients with non-alcoholic fatty liver disease with or without diabetes: An updated systematic review of randomized controlled trials. Diabetes Metab..

[8] Cusi K. (2020). A diabetologist’s perspective of non-alcoholic steatohepatitis (NASH): Knowledge gaps and future directions. Liver Int..

[9] Armstrong M.J., Gaunt P., Aithal G.P., Barton D., Hull D., Parker R., Hazlehurst J.M., Guo K., Abouda G., LEAN Trial Team (2016). Liraglutide safety and efficacy in patients with non-alcoholic steatohepatitis (LEAN): A multicentre, double-blind, randomised, placebo-controlled phase 2 study. Lancet.

[10] Newsome P.N., Buchholtz K., Cusi K., Linder M., Okanoue T., Ratziu V., Sanyal A.J., Sejling A.S., Harrison S.A., Investigators N.N. (2020). A Placebo-Controlled Trial of Subcutaneous Semaglutide in Nonalcoholic Steatohepatitis. N. Engl. J. Med..

[11] Scheen A.J. (2020). Sodium-glucose cotransporter type 2 inhibitors for the treatment of type 2 diabetes mellitus. Nat. Rev. Endocrinol..

[12] Schernthaner G., Schernthaner-Reiter M.H., Schernthaner G.H. (2016). EMPA-REG and Other Cardiovascular Outcome Trials of Glucose-lowering Agents: Implications for Future Treatment Strategies in Type 2 Diabetes Mellitus. Clin. Ther..

[13] Zelniker T.A., Braunwald E. (2020). Clinical Benefit of Cardiorenal Effects of Sodium-Glucose Cotransporter 2 Inhibitors: JACC State-of-the-Art Review. J. Am. Coll. Cardiol..

[14] Wu J.H., Foote C., Blomster J., Toyama T., Perkovic V., Sundstrom J., Neal B. (2016). Effects of sodium-glucose cotransporter-2 inhibitors on cardiovascular events, death, and major safety outcomes in adults with type 2 diabetes: A systematic review and meta-analysis. Lancet Diabetes Endocrinol..

[15] Zelniker T.A., Braunwald E. (2020). Mechanisms of Cardiorenal Effects of Sodium-Glucose Cotransporter 2 Inhibitors: JACC State-of-the-Art Review. J. Am. Coll. Cardiol..

[16] Zelniker T.A., Wiviott S.D., Raz I., Im K., Goodrich E.L., Furtado R.H.M., Bonaca M.P., Mosenzon O., Kato E.T., Cahn A. (2019). Comparison of the Effects of Glucagon-Like Peptide Receptor Agonists and Sodium-Glucose Cotransporter 2 Inhibitors for Prevention of Major Adverse Cardiovascular and Renal Outcomes in Type 2 Diabetes Mellitus. Circulation.

[17] Hsiang J.C., Wong V.W. (2020). SGLT2 Inhibitors in Liver Patients. Clin. Gastroenterol. Hepatol..

[18] Dougherty J.A., Guirguis E., Thornby K.A. (2021). A Systematic Review of Newer Antidiabetic Agents in the Treatment of Nonalcoholic Fatty Liver Disease. Ann. Pharmacother..

[19] Bolinder J., Ljunggren O., Kullberg J., Johansson L., Wilding J., Langkilde A.M., Sugg J., Parikh S. (2012). Effects of dapagliflozin on body weight, total fat mass, and regional adipose tissue distribution in patients with type 2 diabetes mellitus with inadequate glycemic control on metformin. J. Clin. Endocrinol. Metab..

[20] Ito D., Shimizu S., Inoue K., Saito D., Yanagisawa M., Inukai K., Akiyama Y., Morimoto Y., Noda M., Shimada A. (2017). Comparison of Ipragliflozin and Pioglitazone Effects on Nonalcoholic Fatty Liver Disease in Patients With Type 2 Diabetes: A Randomized, 24-Week, Open-Label, Active-Controlled Trial. Diabetes Care.

[21] Kuchay M.S., Krishan S., Mishra S.K., Farooqui K.J., Singh M.K., Wasir J.S., Bansal B., Kaur P., Jevalikar G., Gill H.K. (2018). Effect of Empagliflozin on Liver Fat in Patients With Type 2 Diabetes and Nonalcoholic Fatty Liver Disease: A Randomized Controlled Trial (E-LIFT Trial). Diabetes Care.

[22] Eriksson J.W., Lundkvist P., Jansson P.A., Johansson L., Kvarnstrom M., Moris L., Miliotis T., Forsberg G.B., Riserus U., Lind L. (2018). Effects of dapagliflozin and n-3 carboxylic acids on non-alcoholic fatty liver disease in people with type 2 diabetes: A double-blind randomised placebo-controlled study. Diabetologia.

[23] Cusi K., Bril F., Barb D., Polidori D., Sha S., Ghosh A., Farrell K., Sunny N.E., Kalavalapalli S., Pettus J. (2019). Effect of canagliflozin treatment on hepatic triglyceride content and glucose metabolism in patients with type 2 diabetes. Diabetes Obes. Metab..

[24] Latva-Rasku A., Honka M.J., Kullberg J., Mononen N., Lehtimaki T., Saltevo J., Kirjavainen A.K., Saunavaara V., Iozzo P., Johansson L. (2019). The SGLT2 Inhibitor Dapagliflozin Reduces Liver Fat but Does Not Affect Tissue Insulin Sensitivity: A Randomized, Double-Blind, Placebo-Controlled Study With 8-Week Treatment in Type 2 Diabetes Patients. Diabetes Care.

[25] Shimizu M., Suzuki K., Kato K., Jojima T., Iijima T., Murohisa T., Iijima M., Takekawa H., Usui I., Hiraishi H. (2019). Evaluation of the effects of dapagliflozin, a sodium-glucose co-transporter-2 inhibitor, on hepatic steatosis and fibrosis using transient elastography in patients with type 2 diabetes and non-alcoholic fatty liver disease. Diabetes Obes. Metab..

[26] Kahl S., Gancheva S., Strassburger K., Herder C., Machann J., Katsuyama H., Kabisch S., Henkel E., Kopf S., Lagerpusch M. (2020). Empagliflozin Effectively Lowers Liver Fat Content in Well-Controlled Type 2 Diabetes: A Randomized, Double-Blind, Phase 4, Placebo-Controlled Trial. Diabetes Care.

[27] Johansson L., Hockings P.D., Johnsson E., Dronamraju N., Maaske J., Garcia-Sanchez R., Wilding J.P.H. (2020). Dapagliflozin plus saxagliptin add-on to metformin reduces liver fat and adipose tissue volume in patients with type 2 diabetes. Diabetes Obes. Metab..

[28] Taheri H., Malek M., Ismail-Beigi F., Zamani F., Sohrabi M., Reza Babaei M., Khamseh M.E. (2020). Effect of Empagliflozin on Liver Steatosis and Fibrosis in Patients With Non-Alcoholic Fatty Liver Disease Without Diabetes: A Randomized, Double-Blind, Placebo-Controlled Trial. Adv. Ther..

[29] Han E., Lee Y.H., Lee B.W., Kang E.S., Cha B.S. (2020). Ipragliflozin Additively Ameliorates Non-Alcoholic Fatty Liver Disease in Patients with Type 2 Diabetes Controlled with Metformin and Pioglitazone: A 24-Week Randomized Controlled Trial. J. Clin. Med..

[30] Kinoshita T., Shimoda M., Nakashima K., Fushimi Y., Hirata Y., Tanabe A., Tatsumi F., Hirukawa H., Sanada J., Kohara K. (2020). Comparison of the effects of three kinds of glucose-lowering drugs on non-alcoholic fatty liver disease in patients with type 2 diabetes: A randomized, open-label, three-arm, active control study. J. Diabetes Investig..

[31] Lai L.L., Vethakkan S.R., Nik Mustapha N.R., Mahadeva S., Chan W.K. (2020). Empagliflozin for the Treatment of Nonalcoholic Steatohepatitis in Patients with Type 2 Diabetes Mellitus. Dig. Dis. Sci..

[32] Gallo S., Calle R.A., Terra S.G., Pong A., Tarasenko L., Raji A. (2020). Effects of Ertugliflozin on Liver Enzymes in Patients with Type 2 Diabetes: A Post-Hoc Pooled Analysis of Phase 3 Trials. Diabetes Ther..

[33] Li B., Wang Y., Ye Z., Yang H., Cui X., Wang Z., Liu L. (2018). Effects of Canagliflozin on Fatty Liver Indexes in Patients with Type 2 Diabetes: A Meta-analysis of Randomized Controlled Trials. J. Pharm. Pharm. Sci..

[34] Raj H., Durgia H., Palui R., Kamalanathan S., Selvarajan S., Kar S.S., Sahoo J. (2019). SGLT-2 inhibitors in non-alcoholic fatty liver disease patients with type 2 diabetes mellitus: A systematic review. World J. Diabetes.

[35] McMurray J.J.V., Solomon S.D., Inzucchi S.E., Kober L., Kosiborod M.N., Martinez F.A., Ponikowski P., Sabatine M.S., Anand I.S., Belohlavek J. (2019). Dapagliflozin in Patients with Heart Failure and Reduced Ejection Fraction. N. Engl. J. Med..

[36] Scheen A.J. (2019). Beneficial effects of SGLT2 inhibitors on fatty liver in type 2 diabetes: A common comorbidity associated with severe complications. Diabetes Metab..

[37] Ji W., Zhao M., Wang M., Yan W., Liu Y., Ren S., Lu J., Wang B., Chen L. (2017). Effects of canagliflozin on weight loss in high-fat diet-induced obese mice. PLoS ONE.

[38] Swe M.T., Thongnak L., Jaikumkao K., Pongchaidecha A., Chatsudthipong V., Lungkaphin A. (2019). Dapagliflozin not only improves hepatic injury and pancreatic endoplasmic reticulum stress, but also induces hepatic gluconeogenic enzymes expression in obese rats. Clin. Sci..

[39] Caussy C., Reeder S.B., Sirlin C.B., Loomba R. (2018). Noninvasive, Quantitative Assessment of Liver Fat by MRI-PDFF as an Endpoint in NASH Trials. Hepatology.

[40] Stine J.G., Munaganuru N., Barnard A., Wang J.L., Kaulback K., Argo C.K., Singh S., Fowler K.J., Sirlin C.B., Loomba R. (2020). Change in MRI-PDFF and Histologic Response in Patients with Nonalcoholic Steatohepatitis: A Systematic Review and Meta-Analysis. Clin. Gastroenterol. Hepatol..

[41] Bril F., Barb D., Lomonaco R., Lai J., Cusi K. (2020). Change in hepatic fat content measured by MRI does not predict treatment-induced histological improvement of steatohepatitis. J. Hepatol..

[42] Taylor R.S., Taylor R.J., Bayliss S., Hagstrom H., Nasr P., Schattenberg J.M., Ishigami M., Toyoda H., Wai-Sun Wong V., Peleg N. (2020). Association Between Fibrosis Stage and Outcomes of Patients With Nonalcoholic Fatty Liver Disease: A Systematic Review and Meta-Analysis. Gastroenterology.

[43] Targher G., Byrne C.D., Lonardo A., Zoppini G., Barbui C. (2016). Non-alcoholic fatty liver disease and risk of incident cardiovascular disease: A meta-analysis. J. Hepatol..

[44] Mantovani A., Petracca G., Beatrice G., Tilg H., Byrne C.D., Targher G. (2020). Non-alcoholic fatty liver disease and risk of incident diabetes mellitus: An updated meta-analysis of 501 022 adult individuals. Gut.

[45] Mantovani A., Zaza G., Byrne C.D., Lonardo A., Zoppini G., Bonora E., Targher G. (2018). Nonalcoholic fatty liver disease increases risk of incident chronic kidney disease: A systematic review and meta-analysis. Metabolism.

[46] Lonardo A., Nascimbeni F., Ballestri S., Fairweather D., Win S., Than T.A., Abdelmalek M.F., Suzuki A. (2019). Sex Differences in Nonalcoholic Fatty Liver Disease: State of the Art and Identification of Research Gaps. Hepatology.

[47] Markham A., Elkinson S. (2014). Luseogliflozin: First global approval. Drugs.

[48] Higgins J.P.T., Green S. (2011). Cochrane Handbook for Systematic Reviews of Interventions.

[49] Wan X., Wang W., Liu J., Tong T. (2014). Estimating the sample mean and standard deviation from the sample size, median, range and/or interquartile range. BMC Med. Res. Methodol..

[50] Higgins J.P.T., Green S. Imputing Standard Deviations for Changes from Baseline, Cochrane Handbook for Systematic Reviews of Interventions. http://handbook-5-1.cochrane.org/chapter_16/16_1_3_2_imputing_standard_deviations_for_changes_from_baseline.htm.

[51] Higgins J.P., Thompson S.G. (2002). Quantifying heterogeneity in a meta-analysis. Stat. Med..

[52] Begg C.B., Mazumdar M. (1994). Operating characteristics of a rank correlation test for publication bias. Biometrics.

