# Supplementary material for: Sodium-Glucose Cotransporter-2 Inhibitors for Treatment of Nonalcoholic Fatty Liver Disease: A Meta-Analysis of Randomized Controlled Trials
Source: Metabolites. 2020 Dec 30;11(1):22. doi: 10.3390/metabo11010022 (PMC7823667; doi:10.3390/metabo11010022)
Supplement: Supplementary file 1 [file metabolites-11-00022-s001.pdf]

Online-Only Supplementary Materials

Supplementary Table S1. Clinical trials excluded at the eligibility step of the PRISMA diagram.

Author(s), Reference	Study characteristics	Main reasons for exclusion
Lai et al. (31)	Single-arm, open-label, pilot study: 9 patients with biopsy-proven NASH and T2DM were given empagliflozin 25 mg daily for 24 weeks (*not* placebo-controlled trial)	Unsatisfactory study design
Gallo et al. (32)	Pooled data from seven randomized, double-blind, VERTIS phase-3 controlled trials that evaluated ertugliflozin (5 mg and 15 mg/day) *versus* non-ertugliflozin treatment (placebo, glimepiride, or sitagliptin) in patients with T2DM (irrespective of NAFLD status)	Unsatisfactory study design

Supplementary Table S2. Placebo-controlled or active-controlled RCTs of different SGLT-2 inhibitors for treatment of NAFLD (ordered by publication year).

**Author(s), Year, Reference, Country, Trial name**	**Population, Demographics**	**Interventions (group sizes), Duration (weeks)**	**Efficacy/effectiveness Outcomes A *vs.* B (*vs.* C)**	**Adverse Effects**
Bolinder et al, 2012 (19), International	Patients with type 2 diabetes (inadequately controlled on metformin), and NAFLD (assessed by MRI-PDFF)<br><br>Age: 58.6 y<br><br>Sex: 46.7% male<br><br>Ethnicity: 100% White<br><br>BMI: 31.9 kg/m^2<br><br>HbA1c 7.2%<br><br>Mean ALT and AST: NR	A. Placebo (*n* = 91)<br><br>B. Dapagliflozin (*n* = 91)<br><br>Duration: 24 weeks<br><br>In a subset of patients (*n* = 42 in the placebo arm and n = 38 in the active drug arm), MRI-PDFF was also performed	At week 24, placebo-corrected changes with dapagliflozin were as follows: body weight -2.08 kg, 95% CI -2.84 to -1.31; $p < 0.0001$; total fat mass, -1.48 kg, 95% CI -2.22 to -0.74; $p < 0.0001$<br><br><u>In the MR sub-study</u>: Dapagliflozin produced significantly greater mean reductions from baseline in visceral adipose tissue compared with placebo at 24-week<br><br>Change from baseline at 24-week in mean percent MRI-PDFF fat content with dapagliflozin was −2.35% and −1.53% with placebo, resulting in a not significant placebo-corrected difference of -0.82% (95% CI = -2.97 to 1.33; p = 0.45)	Serious AEs were reported in 6.6% of patients treated with dapagliflozin and in 1.1% of those allocated in placebo group<br><br>Events suggestive of vulvovaginitis, balanitis, and related genital infections were observed in 3.3% of patients treated with dapagliflozin and in none of those allocated in the placebo group
Ito et al, 2017 (20), Multicenter, Japan	Patients with poorly controlled type 2 diabetes and NAFLD (assessed by computed tomography)<br><br>Age: 58 y<br><br>Sex: 48.5% male<br><br>BMI: 30 kg/m^2<br><br>HbA1c 8.4%	A. Pioglitazone (*n* = 34)<br><br>B. Ipragliflozin (*n* = 32)<br><br>Duration: 24 weeks	Mean liver-to-spleen attenuation ratio on computed tomography at week 24 increased by 0.22 (from 0.80 ± 0.24 to 1.0 ± 0.18) in the ipragliflozin group and 0.21 (from 0.78 ± 0.26 to 0.98±0.16) in the pioglitazone group (p = 0.90)<br><br>Liver enzyme levels, HbA1c and HOMA-IR were similarly reduced in the two treatment groups<br><br>FIB4 score was similarly reduced in the two treatment groups	Serious AEs: NR

	Mean ALT 55 IU/mL, AST 41 IU/mL		Body weight and visceral fat area showed significant reductions only in the ipragliflozin group compared with the pioglitazone group	
Kuchay et al, 2018 (21), India, E-LIFT trial	Patients with type 2 diabetes and NAFLD (assessed by MRI-PDFF)<br><br>Age: 50 y<br><br>Sex: 60% male<br><br>BMI: 29.5 kg/m^2<br><br>HbA1c: 9%<br><br>Mean ALT 64 IU/L, AST 44 IU/L	A. Placebo (*n* = 25)<br><br>B. Empagliflozin (*n* = 25)<br><br>Duration: 20 weeks	Empagliflozin was significantly better at reducing liver fat content (mean MRI-PDFF difference between the empagliflozin and control groups 24.0%; $p < 0.0001$)<br><br>Compared with baseline, significant reduction was found in the end-of-treatment MRI-PDFF for the empagliflozin group (16.2% to 11.3%; $p < 0.0001$) and a nonsignificant change was found in the control group (16.4% to 15.5%; $p = 0.057$)<br><br>The two groups showed significant differences for change in serum ALT ($p = 0.005$) and nonsignificant differences for serum AST ($p = 0.212$) and GGT ($p = 0.057$) levels	Serious AEs: NR<br><br>In the empagliflozin group, 22 patients completed the study, with 3 developing AEs related to the study medication<br><br>In the placebo group, 20 patients completed the study, with three lost to follow-up and two patients discontinuing because of work schedule conflicts
Eriksson et al, 2018 (22), Multicenter, Sweden, EFFECT-II trial	Patients with type 2 diabetes and NAFLD (assessed by MRI-PDFF)<br><br>Age: 65.5 y<br><br>Sex: 70% male<br><br>BMI: 31.2 kg/m^2<br><br>HbA1c: 7.5%<br><br>Mean ALT and AST: NR	A. Placebo (*n* = 21)<br><br>B. Omega-3 carboxylic acids (OM-3CA) (*n* = 20)<br><br>C. Dapagliflozin (*n* = 21)<br><br>D. Omega-3 carboxylic acids + dapagliflozin 10 mg/d (*n* = 22)<br><br>Duration: 12 weeks	All active treatments significantly reduced liver fat content from baseline; relative changes were: OM-3CA, −15%; dapagliflozin, −13%; OM-3CA + dapagliflozin, −21%<br><br>Only the combination treatment reduced liver fat content ($p = 0.046$) and total liver fat volume (relative change, −24%, $p = 0.037$) in comparison with placebo<br><br>Dapagliflozin monotherapy, but not the combination with OM-	All active treatment groups had similar total percentages of AE reporting (70.0–77.3%), which were higher than in the placebo group (47.6%)<br><br>More participants reported AEs when using dapagliflozin and OM-3CA (*n* = 15, 68.2%) than when using dapagliflozin monotherapy (*n*= 7, 33.3%), OM-3CA monotherapy (*n* = 8, 40%) or placebo (*n*= 6, 28.6%)

			3CA, reduced serum AST, ALT and GGT levels<br><br>Dapagliflozin alone and in combination with OM-3CA improved HbA1c and reduced body weight	
Cusi et al, 2019 (23), United States	Patients with type 2 diabetes who were unable to maintain glycemic control (most patients had NAFLD on MRI-PDFF)<br><br>Age: 58 y<br><br>Sex: 66% male<br><br>Ethnicity: 67% White<br><br>BMI: 32 kg/m²<br><br>HbA1c 7.7%<br><br>Mean ALT 30 IU/mL, AST 25 IU/mL	A. Placebo (*n* = 30)<br><br>B. Canagliflozin (*n* = 26)<br><br>Duration: 24 weeks	A not significant decrease in liver fat content occurred with canagliflozin (liver fat content: −4.6%, 95% CI −6.4 to −2.7) vs. placebo (−2.4%, 95% CI −4.2 to −0.6, p=0.09). In patients with NAFLD, the decrease in liver fat content was −6.9% (−9.5; −4.2) vs. −3.8% (−6.3; −1.3; p=0.05), respectively<br><br>Body weight loss ≥5% with a ≥30% relative reduction in liver fat content occurred more often with canagliflozin (38% vs. 7%, p=0.009)<br><br>Canagliflozin reduced HbA1c (placebo-subtracted change: −0.71% [−1.08; −0.33]) and body weight (−3.4% [−5.4; −1.4]; both p<0.001)	Serious AEs: NR<br><br>SAE: 3% in placebo group vs. 4% in canagliflozin group
Latva-Rasku et al, 2019 (24), Sweden	Patients with type 2 diabetes and NAFLD (assessed by MRS)<br><br>Age: 60 y<br><br>Sex: 80% male<br><br>Ethnicity: 100% White<br><br>BMI: 32 kg/m²<br><br>HbA1c: 6.9%	A. Dapagliflozin (*n* = 15)<br><br>B. Placebo (*n* = 16)<br><br>Duration: 8 weeks	At week 8, liver fat content was significantly reduced in the dapagliflozin group (from 22 ± 11% at baseline to 18 ± 11% at week 8), but not in the placebo group (from 21 ± 9% at baseline to 21 ± 9% at week 8)<br><br>Dapagliflozin resulted in significant changes in visceral adipose tissue by -0.35 L (95% CI −0.59 to −0.12, *p* < 0.01).<br><br>Serum ALT and AST levels did not significantly decreased in the	Severe AEs: NR

	Mean ALT 44 IU/L, AST 31 IU/L		dapagliflozin group (ALT: from 50±21 IU/L at baseline to 45 ± 16 IU/L at week 8; AST: 30 ± 10 IU/L at baseline to 30 ± 10 IU/L at week 8), and in the placebo group (ALT from 38 ± 14 IU/L at baseline to 39 ± 15 IU/L at week 8; AST from 32 ± 12 IU/L at baseline to 31 ± 10 IU/L at week 8)<br><br>Body mass index significantly decreased in the dapagliflozin group (from 32.1 ± 3.9 kg/m^2 at baseline to 31.3 ± 3.7 kg/m^2 at week 8), but not in the placebo group (from 31.7 ± 5.0 kg/m^2 at baseline to 31.8 ± 4.8 kg/m^2 at week 8)	
Shimitzu et al, 2019 (25), Japan	Patients with type 2 diabetes and NAFLD (assessed by Fibroscan® and CAP measurement)<br><br>Age: 56 y<br><br>Sex<br><br>BMI: 28.0 kg/m^2<br><br>HbA1c: 7.8%<br><br>Mean ALT 36 IU/L, AST 27 IU/L	A. Placebo (n = 24)<br><br>B. Dapagliflozin (n = 33)<br><br>Duration: 24 weeks	In week 24, there was a significant decrease in controlled attenuation parameter (CAP) from 314 ± 61 to 290 ± 73 dB/m ($p = 0.042$) in the dapagliflozin group, but not in the control group<br><br>Liver stiffness measurement (LSM) tended to decrease from 9.49 ± 6.1 to 8.01 ± 5.8 kPa in the dapagliflozin group. In 14 patients from this group with LSM values ≥8.0 kPa, LSM decreased significantly from 14.7 ± 5.7 to 11.0 ± 7.3 kPa ($p = 0.016$)<br><br>Serum ALT and GGT levels decreased significantly in the dapagliflozin group, but not in the control group<br><br>Changes in BMI and HbA1c in the control vs. dapagliflozin groups were: 0.0 (95% CI −0.55 to 0.50) vs. −0.8 (95% CI −1.25 to −0.07) kg/m^2; and HbA1c − 0.3 (95% CI −0.5 to	NR

			0.5) vs. −0.8 (95% CI −1.3 to −0.5)%, respectively	
Kahl et al, 2020 (26), Germany	Patients with well-controlled type 2 diabetes and NAFLD (assessed by MRS)<br><br>Age: 62 y<br><br>Sex: 69% male<br><br>Ethnicity: 100% White<br><br>BM: 32.2 kg/m^2<br><br>HbA1c 6.6%<br><br>Mean ALT 35 IU/mL, AST 25 IU/mL	A. Placebo (*n* = 42)<br><br>B. Empagliflozin 25 mg/d (*n* = 42)<br><br>Duration: 24 weeks	Empagliflozin treatment resulted in a placebo-corrected absolute of 21.8% (95% CI 23.4, 20.2%; *p* = 0.02) and relative change in liver fat content of -22% (-36, -7%; *p* = 0.009) from baseline to end of treatment, corresponding to a 2.3-fold greater reduction<br><br>Weight loss occurred only with empagliflozin (placebo-corrected change -2.5 kg [-3.7, -1.4 kg]; *p* < 0.001), while no placebo-corrected change in tissue-specific insulin sensitivity was observed<br><br>Serum ALT and GGT levels were reduced with similar effect sizes between the empagliflozin and placebo groups at 24 weeks	Serious AEs: NR<br><br>All treatment groups had similar percentages of AEs (5 events in the empagliflozin group and 7 events in the placebo group)
Johansson et al, 2020 (27), International	Patients with T2DM, treated with metformin ≥1500 mg/day for at least 8 weeks, and NAFLD (assessed by MRI-PDFF)<br><br>Age: 58 y<br><br>Sex: 50% male<br><br>Ethnicity: 100% White<br><br>BMI: 32.6 kg/m^2<br><br>HbA1c: 8.5%<br><br>Mean ALT 30 IU/L, AST 25 IU/L	A. Glimepiride + metformin (*n* = 36)<br><br>B. Dapagliflozin + saxagliptin+ metformin (*n* = 46)<br><br>Duration: 52 weeks	At week 52, dapagliflozin, co-administered with saxagliptin and metformin, significantly decreased liver fat content (MRI-PDFF: from 14.3 ± 6.4% at baseline to 9.9 ± 7.1% at week 52), visceral adipose tissue volume (from 3.6 ± 1.1 L at baseline to 3.2 ± 1.1 L at week 52) and body weight (from 90.8 ± 19 kg at baseline to 88.4 ± 18 kg at week 52) vs. glimepiride *plus* metformin (MRI-PDFF: from 13.7 ± 8.3% at baseline to 12.9 ± 8.6% at week 52; visceral adipose tissue volume: from 2.9 ± 1.1 L at baseline to 3.0 ± 1.1 L at week 52; body weight: from 88.4 ± 17 kg at baseline to 90.6 ± 17 kg at week 52)<br><br>At week 52, there was a decrease from baseline in serum	Serious AEs: NR<br><br>All treatment groups had similar percentages of AEs

			aminotransferases in the dapagliflozin *plus* saxagliptin *plus* metformin treatment group (ALT from 30 ± 15 IU/L at baseline to 25 ± 15 IU/L at week 52; AST from 25 ± 10 IU/L to 23 ± 10 IU/L at week 52) compared with glimepiride *plus* metformin treatment group (ALT from 30 ± 15 IU/L at baseline to 32 ± 15 IU/L at week 52; AST from 25 ± 10 IU/L to 27 ± 10 IU/L at week 52)	
Taheri et al, 2020 (28), Iran	Nondiabetic patients with NAFLD (on Fibroscan® associated with CAP measurement)<br><br>Age: 44 y<br><br>Sex: 56% male<br><br>Ethnicity: 100% Arabian<br><br>BMI: 30.5 Kg/m^2<br><br>HbA1c: NR<br><br>Mean ALT 36 IU/L, AST 26 IU/L	A. Placebo (*n* =47)<br><br>B. Empagliflozin 10 mg/d (*n* = 43)<br><br>Duration: 24 weeks	In week 24, there was a significant decrease in CAP in the empagliflozin group (from 306.5 ± 24 to 277.7 ± 32 dB/m, *p* = 0.001) and in the control group (from 304.6 ± 27 to 281.2 ± 35 dB/m, *p* = 0.001)<br><br>Liver stiffness measurement significantly decreased from 6.03 ± 1.4 to 5.33 ± 1.1 kPa (*p* = 0.001) in the empagliflozin group, but not in the control group (from 5.56 ± 1.0 to 5.35 ± 0.96 kPa, *p* = 0.139)<br><br>Serum ALT and AST levels significantly decreased in the empagliflozin group (ALT from 39.1 ± 24 to 32.3 ± 18 IU/L, *p* = 0.007; AST from 25.8 ± 10 to 22.4 ± 7 IU/L, *p* = 0.004), but not in the control group (ALT from 33.4 ± 21 to 31.8 ± 20 IU/L, *p* = 0.545; AST from 24.8 ± 9 to 23.6 ± 9 IU/L, *p* = 0.385)<br><br>BMI significantly decreased in the empagliflozin group (from 30.5 ± 2.3 to 29.9 ± 2.8 kg/m^2, *p* = 0.002), but not in the control group (from	Serious AE: NR<br><br>Mild fungal vaginal infections were reported in 2 patients in the empagliflozin group and 3 patients in the placebo group

			30.7 ± 3.5 to 30.9 ± 3.8 kg/m², *p* = 0.201)	
Han et al, 2020 (29), South Korea	Patients with type 2 diabetes and NAFLD (on Fibroscan® associated with CAP measurement)<br><br>Age: 55 y<br><br>Sex: 62% male<br><br>Ethnicity: 100% Asian<br><br>BMI: 30.3 Kg/m²<br><br>HbA1c: 6.6%<br><br>Mean ALT 32 IU/L, AST 28 IU/L	A. Metformin + pioglitazone (*n* =15)<br><br>B. Metformin + pioglitazone + ipragliflozin (*n* = 30)<br><br>Duration: 24 weeks	At week 24, ipragliflozin was associated with reduced liver fat content (CAP from 306.6 ± 40 to 298.6 ± 45 dB/m). This was not observed in the control group (CAP from 307.7 ± 37 dB/m at baseline to 319.5 ± 45 dB/m at week 24). Ipragliflozin also reduced visceral adipose tissue (from 209.1 ± 63 cm² at baseline to 182.9 ± 64 cm² at week 24). This was not observed in the control group (visceral adipose tissue from 223.3 ± 91 cm² at baseline to 230.3 ± 88 cm² at week 24)<br><br>Serum ALT and AST levels decreased in the ipragliflozin add-on group (ALT from 33.4 ± 25 IU/L at baseline to 25.6 ± 17 IU/L at week 24; AST from 26.6.1 ± 13 IU/L at baseline to 24.3 ± 10.6 IU/L at week 24), but also in the control group (ALT from 31.1 ± 14 IU/L at baseline to 26.5 ± 12 IU/L at week 24; AST from 30.4 ± 20 IU/L at baseline to 24.7 ± 10 IU/L at week 24)<br><br>BMI decreased in the ipragliflozin add-on group (from 30.6 ± 5.3 kg/m² at baseline to 30.1 ± 5.3 kg/m² at week 24), but not in the control group (from 30.2 ± 2.5 kg/m² at baseline to 30.4 ± 2.6 kg/m² at week 24)	In the ipragliflozin add-on group, 8 patients exhibited symptoms of hypoglycemia, 11 patients reported renal and urinary disorders and 3 patients had cystitis
Kinoshita et al, 2020 (30), Japan	Patients with type 2 diabetes and NAFLD (assessed by the liver-to-spleen ratio on computed tomography)	A. Dapagliflozin (*n* = 32)<br><br>B. Pioglitazone (*n* = 33)	At week 28, the liver-to-spleen (L/S) ratio was increased in the dapagliflozin group (from 0.75 ± 0.04 at baseline to 0.91 ± 0.05 at week 28); in the pioglitazone	Severe AEs: NR.<br><br>More participants in the pioglitazone group reported peripheral oedema (15%) than in

	Age: 59 y<br><br>Sex: 46% male<br><br>Ethnicity: 100% Asian<br><br>BMI: 28.8 kg/m^2<br><br>HbA1c: 7.5%<br><br>Mean ALT 47 IU/L, AST 35 IU/L	C. Glimepiride (n = 33)<br><br>Duration: 28 weeks	group (from 0.74 ± 0.04 at baseline to 0.96 ± 0.06 at week 28), but not in the glimepiride group (from 0.75 ± 0.04 at baseline to 0.76 ± 0.05 at week 28). Visceral adipose tissue was reduced in the dapagliflozin group (from 193.4 ± 11 cm^2 at baseline to 173.6 ± 9 cm^2 at week 28), but not in the pioglitazone group (from 174.2 ± 13 at baseline to 176.7 ± 12 at week 28), or the glimepiride group (from 169.6 ± 10 cm^2 at baseline to 176.4 ± 10 cm^2 at week 28)<br><br>Serum ALT and AST levels decreased in the dapagliflozin group (ALT from 50.3 ± 4.7 IU/L at baseline to 37.4 ± 4.5 IU/L at week 24; AST from 38.8 ± 4.1 IU/L at baseline to 30.2 ± 3.1 IU/L at week 24), and in the pioglitazone group (ALT from 46.1 ± 6.1 IU/L at baseline to 31.0 ± 3.5 IU/L at week 24; AST from 34.1 ± 3.9 IU/L at baseline to 26.9 ± 2.0 IU/L at week 24), but not in the glimepiride group (ALT from 45.3 ± 4.6 IU/L at baseline to 44.3 ± 4.7 IU/L at week 24; AST from 32.3 ± 2.5 IU/L at baseline to 32.7 ± 2.6 IU/L at week 24)<br><br>Body weight decreased in the dapagliflozin group (from 77.1 ± 2.9 kg at baseline to 74.3 ± 3.0 kg at week 24), but not in the pioglitazone group (from 75.7 ± 2.7 kg at baseline to 77.1 ± 2.8 kg at week 24) and in the glimepiride group (from 75.0 ± 3.3 kg at baseline to 77.5 ± 3.5 kg at week 24)	the dapagliflozin (0%) and glimepiride (0%) groups

Abbreviations: AEs, adverse effects; ALT, alanine aminotransferase; AST, aspartate aminotransferase; BMI, body mass index; MRI-PDFF, magnetic resonance imaging-proton density fat fraction; MRS, magnetic resonance spectroscopy; NAFLD, non-alcoholic fatty liver disease; NASH, non-alcoholic steatohepatitis; NR, not reported.

Supplementary Table S3. Risk of bias for each RCT assessed by the Cochrane Collaboration's tool.

Author(s)	Year	Random sequence generation (selection bias)	Allocation concealment (selection bias)	Blinding of participants and personnel (performance bias)	Blinding of outcome assessment (detection bias)	Incomplete outcome data (attrition bias)	Selective reporting (reporting bias)	Other Bias *
**Bolinder et al.**	2012	Low	Low	Low	Low	Unclear	Low	Unclear
**Ito et al.**	2017	Low	Unclear	Low	Low	Low	Low	High
**Kuchay et al.**	2018	Low	Low	Low	Low	Low	Low	Unclear
**Eriksson et al.**	2018	Low	Unclear	Low	Low	Unclear	Low	Unclear
**Cusi et al.**	2019	Low	Low	Low	Low	Unclear	Low	Unclear
**Latva-Rasku et al.**	2019	Low	Low	Low	Low	Low	Low	Unclear
**Shimitzu et al.**	2019	Low	Low	Low	Low	Low	Low	High
**Kahl et al.**	2020	Low	Low	Low	Low	Unclear	Low	Unclear
**Johansson et al.**	2020	Low	Unclear	Low	Low	Unclear	Low	Unclear
**Taheri et al.**	2020	Low	Unclear	Low	Low	Low	Low	High
**Han et al.**	2020	Low	Low	Low	Low	Low	Low	High
**Kinoshita et al.**	2020	Low	Low	Low	Low	Unclear	Low	High

* Note: for each of the seven domains of the Cochrane Collaboration's tool the presence of low risk of bias was highlighted in green; unclear risk was highlighted in yellow, and high risk of bias was highlighted in red. Since there were no published RCTs with paired liver biopsy data (i.e., the reference method for assessing drug-induced changes in hepatic steatosis, necro-inflammation or fibrosis), we arbitrarily assigned an unclear risk of bias in the "Other Bias" domain of the Cochrane Collaboration's tool when RCTs used MRI-PDFF or MRS, or a high risk of bias when RCTs used computed tomography or CAP measurement on Fibroscan®.

PRISMA 2009 Flow Diagram

Identification

Records identified through database searching on PubMed, Scopus and ClinicalTrials.Gov up to October 31, 2020
(n = 29)

Additional records identified through other sources
(n = 2)

Screening

Records after duplicates removed
(n = 30)

Records screened
(n = 30)

Records excluded
(n = 16)

Eligibility

Full-text articles assessed for eligibility
(n = 14)

Full-text articles excluded, with reasons
(n = 2)

Included

RCTs included in qualitative synthesis
(n = 12)

RCTs included in quantitative synthesis (meta-analysis)
(n = 12)

From: Moher D, Liberati A, Tetzlaff J, Altman DG, The PRISMA Group (2009). *P*referred *R*eporting *I*tems for *S*ystematic Reviews and *M*eta-*A*nalyses: The PRISMA Statement. PLoS Med 6(6): e1000097. doi:10.1371/journal.pmed1000097

For more information, visit www.prisma-statement.org.

Supplementary Figure S1. The PRISMA flow diagram for search and selection processes of the meta-analysis.

Panel A

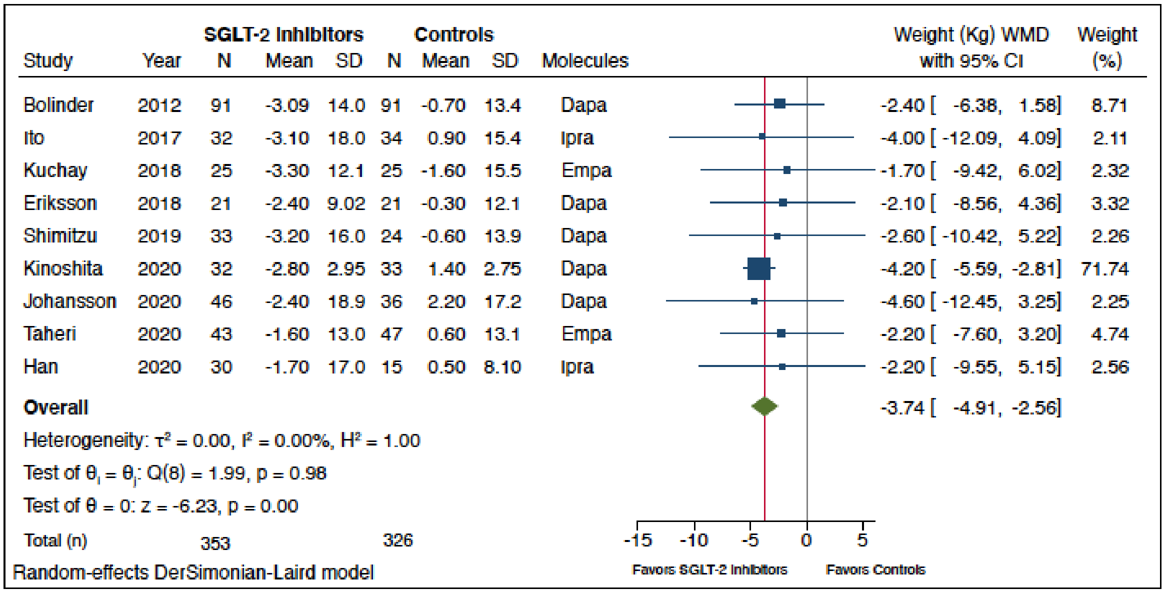

Panel B

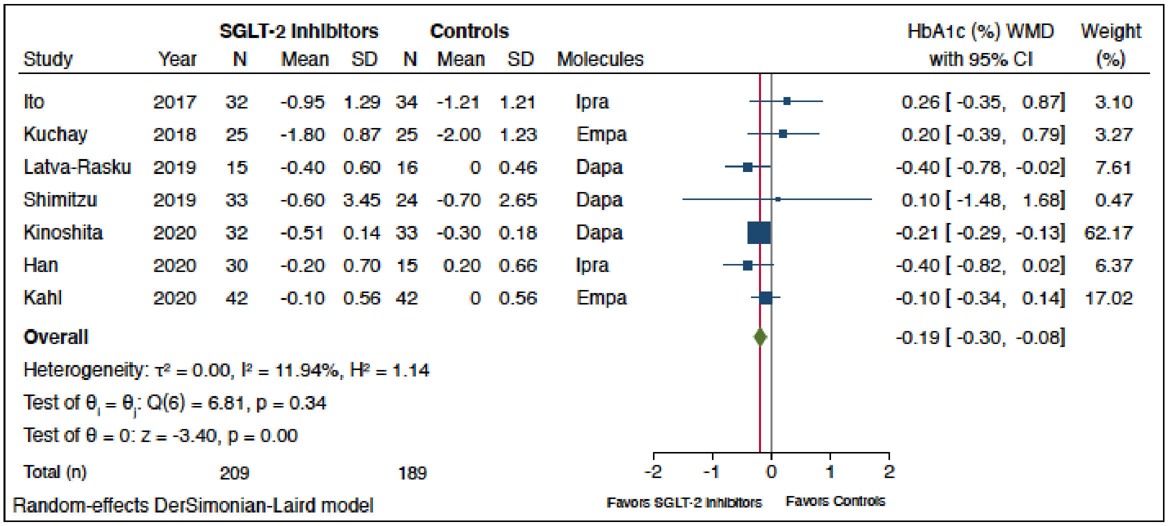

Supplementary Figure S2. Forest plot of the effects of SGLT-2 inhibitors on body weight (*n* = 9 RCTs, panel **A**) and hemoglobin A1c levels (*n* = 7 RCTs, panel **B**) as compared with placebo or reference therapy. The effect size was expressed as weighted mean difference (WMD) and 95% confidence intervals for all RCTs included. Note: If not available, the SDs of the mean differences were estimated using a specific formula (as specified in the Methods section).

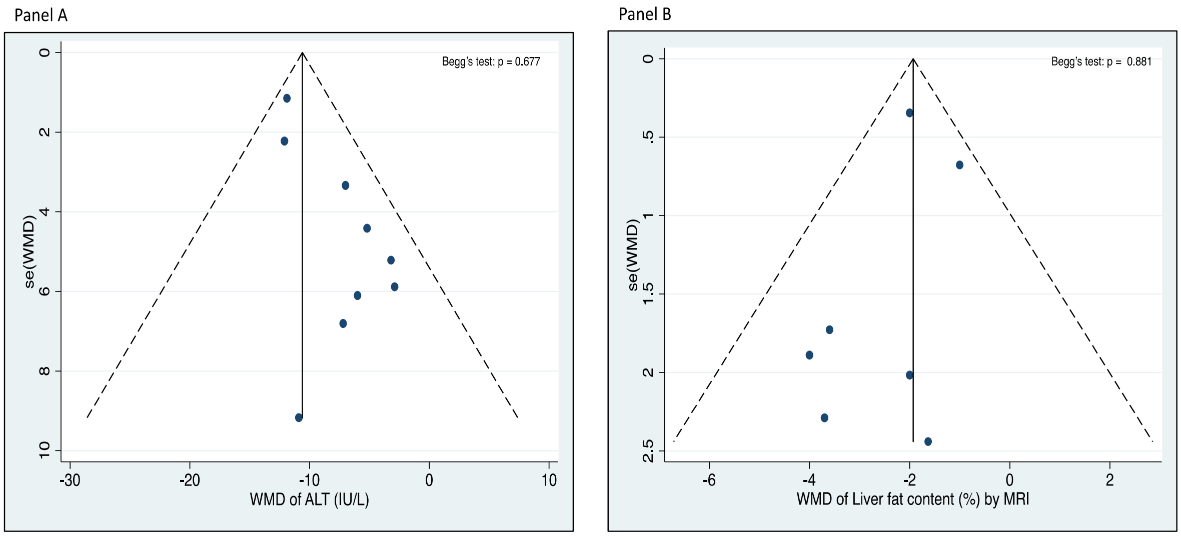

Supplementary Figure S3. Funnel plots of standard errors by weighted mean difference (WMD) in serum ALT levels (n = 9 RCTs, panel **A**) and liver fat content assessed by magnetic resonance-based techniques (n = 7 RCTs, panel **B**). p-values were assessed by the rank correlation Begg's test.
